# Editorial: Improving crop nutritional security for sustainable agriculture in the era of climate change

**DOI:** 10.3389/fpls.2023.1292264

**Published:** 2023-09-25

**Authors:** M. Iqbal R. Khan, Mohammad Irfan, Ravi Gupta

**Affiliations:** ^1^ Department of Botany, Jamia Hamdard, New Delhi, India; ^2^ School of Integrative Plant Science, College of Agriculture and Life Sciences, Cornell University, Ithaca, NY, United States; ^3^ College of General Education, Kookmin University, Seoul, Republic of Korea

**Keywords:** environment, climate change, nutritional security, food security, plant

Global nutritional security is indispensable to boost agricultural productivity to feed the growing population, and amid the challenges posed by climate change. Thus, securing our future food supply remains a critical concern. Several factors including population size, climatic fluctuations, and the environmental degradation causing declination in the quality of natural resources (land, water and air). These factors contribute and lead to the nutritional instabilities, consequently hampering the sustainable production of agricultural goods. Hence, food security has become a major issue in the recent decade and is going to be intensified further in the coming years. Despite signature achievements in the agricultural industry, reports suggest that there are approximately 820 million people who are suffering from chronic hunger, and 2 billion lack a sufficient supply of nutrition in their daily intake of food (Bhardwaj et al., 2022). This duplex burden of malnutrition has been emerging as the ‘new normal’ in the present circumstances. Thus, nutritional security is a subject of major concern from the perspective of human health as well as livestock, while sustaining the agricultural inputs for the burgeoning population size. Researchers have been establishing numerous ways to alleviate nutritional insecurity through conventional and biotechnological interventions, few of these have been discussed here.

## Strategic interventions to improve sustainable nutritional security under environmental stresses

### Plant growth, physiology, and metabolic profile management

In the pursuit of future food security, a critical challenge lies in deciphering how climate variables interact with plant growth, fitness, and yield parameters. Such interactions can trigger unique shifts in various aspects of plants, including morphology, physiology, and metabolite accumulation, ultimately leading to adaptive responses tailored to specific climate scenarios (Nazir et al., 2023). Večeřová et al. deciphered the intricate interactions between climate variables and plant responses. By subjecting spring wheat cultivar Cadenza to simulated climate conditions projected for the year 2100, they uncovered a mitigating effect of elevated carbon dioxide (CO_2_) on the adverse impacts of dynamic changes in the temperature range. Metabolite profiling revealed the accumulation of key compounds linked to cellular energy, biosynthesis, and oxidative balance. This pioneering research demonstrates the potential for developing climate-resilient wheat cultivars by leveraging these identified metabolites as breeding targets, ultimately bolstering food and nutritional security in the face of changing climates. Considering the potential role of metabolites, plant secondary metabolites (SMs) were also found pivotal in modulating the growth and developmental processes, along with exhibiting a variety of therapeutic effects in response to environmental stressors. For instance, in order to understand the medicinal properties of Euphorbia latex, Benjamaa et al. reviewed the therapeutic potential of latex obtained from various Euphorbia species. Although Euphorbia latex is considered poisonous and exhibits toxicity if consumed raw in larger amounts, it has been used in traditional medicines for ages. Euphorbia latex is known to contain various SMs such as phenolic compounds, alkaloids, saponins, and flavonoids, among others that potentially account for its antioxidant, antimicrobial, and a variety of therapeutic effects. In the past, Euphorbia latex has been used for the treatment of various diseases including some of the most serious diseases such as dropsy, paralysis, and deafness, along with some of the common diseases like wounds, skin warts, and amaurosis, among others. In addition, based on recent research, the anti-carcinogenic potential of Euphorbia latex was also highlighted.

### Fertilizer management and its systemic utilization

Over the last few years, research has delved into multifaceted strategies that hold promise for sustainable agriculture under changing climatic conditions. One such approach under the attention is effective fertilizer management, which plays a pivotal role in ensuring crop productivity and nutritional security. Sustainable utilization and management of fertilizers critically determine the agricultural productivity under diverse climatic fluctuations. In this approach, Reduced Basal and Increased Topdressing (RBIT) fertilizer rate has been found as an efficient approach to avoid yield penalties of rice (*Oryza sativa*) and wheat (*Triticum aestivum*) in response to environmental stressors. Zhang et al. explored the potential of RBIT fertilizer rates to address the challenges posed by climate change. Conducted over a span of nine years in the lower Yangtze River rice-wheat system region of China, the study revealed significant enhancements in rice yields. RBIT, when combined with straw incorporation, led to substantial increases in both average and annual rice yields. Notably, this approach displayed a remarkable potential to elevate the Sustainable Yield Index (SYI) of wheat and rice by 7.6% and 12.8%, respectively, compared to conventional fertilization. The implications extend beyond mere yield improvements; RBIT emerged as a potent driver of carbon (C) sequestration, with the combination of RBIT and straw incorporation showing the highest rates of sequestration. This study highlights the positive effects of these practices on soil health, emphasizing their role in fortifying rice production sustainability and soil organic carbon (SOC) sequestration. Accurately assessing the C footprint of rice cultivation is pivotal in curbing greenhouse gas emissions from global food production. To enhance the credibility and efficiency of this evaluation, Xu et al. conducted sensitivity and uncertainty analyses on a C footprint model integrated with a typical East Asian rice production system employing different fertilization approaches. The outcomes of sensitivity and uncertainty analyses shed light on crucial parameters, with methane (CH_4_) emission estimation taking center stage. Notably, organic rice production exhibited a C footprint significantly surpassing that of conventional rice production, mainly attributed to increased CH_4_ emissions impact. This comprehensive framework offers valuable insights for shaping future strategies aimed at mitigating the greenhouse gas effects of rice production.

### Mutagens-mediated approaches to achieve high-yield crop varieties

The development of high-yielding cultivars is another important aspect that must be taken into consideration for achieving food security. Therefore, researchers around the globe are making continuous attempts to generate high-yielding cultivars by a variety of breeding approaches. Raina and Khan utilized γ-rays and sodium azide-based induced mutagenesis to develop high-yielding cowpea (*Vigna unguiculata* L. Walp.) cultivars (Raina and Khan, 2023). The authors showed that mutants developed using low and intermediate doses of these mutagens exhibit improved agronomic traits as compared to their parental lines Gomati VU-89 and Pusa-578. In particular, the number of seeds per pod was highly increased among other agronomic traits; however, these mutations led to a decline in the overall plant height, the contents of chlorophyll and carotenoids, and the activity of nitrate reductase (NR) enzymes. These results indicate that plant growth parameters and agronomic traits are negatively correlated and targeting one may compromise the other characteristics in cowpea.

### Transcriptional regulations-mediated strategies

Amidst the changing landscape of nutritional security and climate change, the exploration of plant defence mechanisms gains prominence (Iqbal et al., 2021). The enigmatic role of WRKY transcription factors (TFs) emerges as a pivotal aspect of ensuring both crop productivity and food security in the countenance of evolving climate patterns. In a bid to decipher the intricate interplay between genetic factors, climate variations, and disease resistance, Yan et al. delve into *WRKY* family TFs functions in peanuts (*Arachis hypogaea*). The study unveils 174 *WRKY* genes (*AhWRKY*) within the peanut genome, offering a deeper understanding of their genetic composition and potential functions. Moreover, this study also investigates how these *WRKY* genes respond to *Ralstonia solanacearum* infection, a prominent disease affecting peanuts. Ankit et al. utilized an integrated morpho-physiological and transcriptome analysis to understand the effects of potassium (K^+^) deficiency on the chickpea cultivars {Formatting Citation}. The authors compared PUSA362 (K^+^ deficiency-sensitive) and PUSA372 (K^+^ deficiency-tolerant) cultivars of chickpea of which PUSA362 showed stunted plant growth along with reduced root development under K^+^ deficiency while PUSA372 was less affected. RNA-seq-based transcriptome analysis identified 820 K^+^ deficiency-responsive genes that were majorly associated with metabolism, signal transduction, TFs, ion transport, phytohormones, and root development. In particular, the sensitive cultivar showed suppression of genes associated with sucrose transport from shoot to root, indicating a lower energy production and thus contributing to the reduced root growth in PUSA362. Based on these results, authors concluded that the interplay of these genes, particularly those related to phytohormones, signal transduction, ion transport, and TFs, contributes to the differential response to K^+^ deficiency in chickpea.

### The interplay between plant signaling regulators

A review by Son and Park highlighted the effects of varying climatic factors such as temperature, humidity, and high CO_2_ on plant immune responses. In addition, the authors also reviewed the effects of endogenous calcium (Ca^2+^) ions and hydrogen peroxide (H_2_O_2_) concentrations on plant defence against invading pathogens. Plant immunity consists of two major components, pathogen-associated molecular pattern (PAMP)-triggered immunity (PTI) and effector-triggered immunity (ETI; Meng et al., 2019). Of these, PTI acts as a first line of defence and is activated upon recognition of conserved pathogen-derived molecular signatures that triggers a rapid oxidative burst, callose deposition, and closure of stomata to inhibit the entry of invading pathogens inside the plant cells (Gupta et al., 2015). On the other hand, ETI is comparatively more robust signaling that may lead to a hypertensive response to kill the pathogen-infected cells. Phytohormone, salicylic acid (SA), has been shown to play key roles in the activation of plant immune responses and the effects of temperature and other environmental and endogenous factors on the SA-dependent and -independent immune responses was reviewed. In conclusion, these changing environmental factors are shown to repress the plant immune responses and affect global food security negatively.

In summary, these studies collectively highlight innovative approaches and insights that are integral to addressing the intertwined challenges of climate change and nutritional security. From sustainable nutritional management and their footprint assessment to deciphering plant responses and disease resistance mechanisms, these findings offer promising pathways for ensuring a resilient and secure global food supply.

## Green technologies-based sustainable agricultural practices to ensure the nutritional security in environmentally-challenged crops

Green technologies have been emerging as the potential tool to manage the nutritional security to sustain the agricultural productivity in the epoch of unexpected climatic conditions. Considering the present situation, several researchers explored the methodologies to incorporate and align the concept of green technologies with nutritional security. For instance, Li and Lin investigated the effects of green technology application on sustainable grain production and developed threshold models to improve agricultural production in China. In particular, the authors analyzed the impact of green technologies on Green Total Factor Productivity Growth (GTFPG) in the context of agricultural mechanized production. They especially focused on the green technologies related to plowing, sowing, fertilization, and irrigation to construct the threshold models. The authors showed that the correlation between GTFPG and green technologies varies across regions, with distinct preferences for specific technologies in major and non-major grain-producing areas. Based on their results, the authors concluded the importance of tailored agricultural strategies considering regional disparities and technology-specific effects to promote sustainable productivity growth in the agricultural sector in China. Altogether, green nanoscience and nanotechnology has been regarded as the ‘roadmap’ to explore the different facets to achieve the nutritional homeostasis for agricultural sustainability (Rasheed et al., 2022).

Conclusively, this article contributes and comprehends with the present knowledge of nutritional security as a regulatory action to avail the sustainable practices of agriculture. Herein, the food nutritional value serves as the mediator in harmonizing the relationship between the plant and human health, as well as the livestock fidelity. The article has discussed the recent progression and strategic approaches for achieving the underlying aim, however, future studies focusing on these disciplines will help to unravel environmental stress-responsive networks and cascade signaling pathways and elucidate how they act together to determine which pathways may have induced first in the environmental stress, will further aid to create strategies for boosting the current status of food nutrition, which are becoming more widespread and frequent due to the persistent but abrupt climate changes.

## Author contributions

MK: Conceptualization, Writing – original draft. MI: Writing – review & editing. RG: Writing – review & editing.
